# Recovery From Repeat Mild Traumatic Brain Injury in Adolescent Rats Is Dependent on Pre-injury Activity State

**DOI:** 10.3389/fneur.2020.616661

**Published:** 2021-01-08

**Authors:** Lindsay Ferguson, Christopher C. Giza, Rebecka O. Serpa, Tiffany Greco, Michael Folkerts, Mayumi L. Prins

**Affiliations:** ^1^University of California Los Angeles, David Geffen School of Medicine, Department of Neurosurgery, Brain Injury Research Center, Los Angeles, CA, United States; ^2^University of California Los Angeles, Steve Tisch BrainSPORT Program, Los Angeles, CA, United States; ^3^Department of Psychology, Seaver College, Pepperdine University, Malibu, CA, United States

**Keywords:** secondary insult, pediatric brain injury, rat, cognitive function, behavioral assessment

## Abstract

Adolescents and young adults have the highest incidence of mild traumatic brain injury (mTBI); sport-related activities are a major contributor. Roughly a third of these patients diagnosed with mTBI are estimated to have received a subsequent repeat mTBI (rTBI). Previously, animal studies have only modeled mTBI in sedentary animals. This study utilizes physical activity as a dependent variable prior to rTBI in adolescent rats by allowing voluntary exercise in males, establishing the rat athlete (rathlete). Rats were given access to locked or functional running wheels for 10 d prior to sham or rTBI injury. Following rTBI, rathletes were allowed voluntary access to running wheels beginning on different days post-injury: no run (rTBI+no run), immediate run (rTBI+Immed), or 3 day delay (rTBI+3dd). Rats were tested for motor and cognitive-behavioral (anxiety, social, memory) and mechanosensory (allodynia) dysfunction using a novel rat standardized concussion assessment tool on post-injury days 1,3,5,7, and 10. Protein expression of brain derived neurotrophic factor (BDNF) and proliferator-activated gamma coactivator 1-alpha (PGC1α) was measured in the parietal cortex, hippocampus, and gastrocnemius muscle. Sedentary shams displayed lower anxiety-like behaviors compared to rathlete shams on all testing days. BDNF and PGC1α levels increased in the parietal cortex and hippocampus with voluntary exercise. In rTBI rathletes, the rTBI+Immed group showed impaired social behavior, memory impairment in novel object recognition, and increased immobility compared to rathlete shams. All rats showed greater neuropathic mechanosensory sensitivity than previously published uninjured adults, with rTBI+3dd showing greatest sensitivity. These results demonstrate that voluntary exercise changes baseline functioning of the brain, and that among rTBI rathletes, delayed return to activity improved cognitive recovery.

## Introduction

According to the 2017 Center for Disease Control report the overall incidence of traumatic brain injury (TBI) has increased by more than 50% from 534.4 per 100,000 people to 787.1 per 100,000 since 2007 (1). The majority (~80%) of TBI are considered mild (mTBI), with peak incidence occurring among adolescents and young adults (age 15–24) and predominantly through sports or recreational activities (1, 2). Incidence of repeat concussion (rTBI) is difficult to quantify as not all individuals seek medical care. Estimates suggest that up to a third of adolescents with concussions have a history (self-reported and/or diagnosed by onsite athletic trainer) of prior concussions (3–5). Sport-related injuries are the largest (>50%) contributor to rTBI. At the high school level, roughly 50% of students participate in sport-related or highly physical (≥60 min per day) activities, representing a high proportion of individuals at risk for mTBI and rTBI (6). Sedentary individuals are defined by ≥3 hours/day of sedentary behavior (watching television, playing video games) (7). The risk for a subsequent TBI is associated with the number of previous concussions and with age (8, 9). Nearly half of those with mTBI or rTBI are athletes who play contact sports. When current return-to-play guidelines are enforced, risk for secondary injury is reduced and occurs on average 60 days after initial injury (10). This is an improvement from 15 years ago when 90% of rTBI occurred within the first 10 days following initial injury of in-season athletes (11, 12).

TBI is a growing concern for adolescents and young adults as injury may have distinct effects during this time of ongoing cerebral development. Early in puberty individuals undergo a phase of cortical growth followed by synaptic pruning, myelination, and stabilization later in adolescence (13). While cortical developmental plasticity was predicted to protect the young brain from injury, recent studies have shown that stressors, like mTBI, during this time make the adolescent brain more susceptible to their harmful effects (13–15). Morphological changes of the prefrontal cortex have been shown to last longer than adults due to stress as well as decreased social interactions (15). These individuals may be prone to neurological deficits resulting from mTBI during a critical time of development and may lead to long-term health problems, premature mortality, and lower education attainment (16). The adolescent stress response lasts longer than adults and predisposes adolescents to psychological deficits including anxiety and depression (17). Further repeat injuries lead to poorer outcomes (16). Adolescence therefore represents the confluence of complex neurodevelopmental changes, risky behaviors, and more intense physical activities that lead to increased risk of rTBI, with potential for negative long-term consequences. Despite this, the effects of head injury in adolescents are not well-studied.

A unique feature of the adolescent athlete is that they are physically active and physiologically fit compared to sedentary peers. Exercise has known benefits for the body and brain. One study of aerobic exercise on males (15–18 years old) found increased connectivity and microstructure in the corticospinal tract, along with increased academic performance (18, 19). Specifically, high school athletes showed higher grade point average in a variety of studies compared with low fitness adolescents (20). Cardiovascular fitness in children (age 8–11) has also been shown to increase white matter integrity (21). Aging adults also see sustained gray and white matter volume due to exercise (21, 22). Fitness training in adult animals, through running wheel (voluntary) and treadmill (forced) exercise, promotes changes in the brain as well. In rodents, running has been shown to increase mitochondrial activity and synaptic plasticity through upregulation of a variety of proteins related to energy metabolism, cytoskeletal structure, and phosphorylation (23, 24). Studies of juvenile and adult rats show increased dendritic branching, capillary expansion, and neurochemical changes due to exercise. Animal studies employing running in older rats have found increased dopamine receptor density and enhanced choline uptake in frontal and parietal cortices and may help explain the improvement in cognitive function. While both human and animal studies have shown that aerobic fitness is beneficial for cardiovascular and brain health in adults, few have evaluated these relationships in the setting of brain injury (20, 25–27). Importantly, current research models of mTBI in any age group are NOT conducted in exercised, “athlete,” animal models. It remains unclear whether adolescent athletes respond differently to mTBI than sedentary peers.

In this two-part study we first address the health benefits of voluntary cardiovascular exercise on the brain in adolescent male rats, and how rat athletes (rathletes) differ from sedentary rats following rTBI. While voluntary aerobic exercise is known to cause changes to the brain, most animal studies use sedentary animals to study TBI. The current study uses pre-injury exercise to address recovery of rathletes following rTBI. Post-injury measurements of motor function, balance, memory, anxiety, and allodynia were analyzed in adolescent rathletes. The first experiment addresses the question: How does fitness affect the vulnerability of the brain to injury? We hypothesize that exercise will decrease body weight over time, increase BDNF and PGC1α and improve cognitive performance.

Second, we address the issue of return to activity following concussion in the current rathlete model, considering the prior exercised state of the rats. Current protocols for concussed individuals suggest waiting until becoming asymptomatic before returning to activity. Exercise too early following TBI may exacerbate post-concussive symptoms and exacerbate neurocellular dysfunction despite resolution of physical symptoms. However, some studies have found that early exercise following TBI improved cognitive performance and decreased time for symptom resolution (28, 29). The second experiment addresses the following questions: Does regular exercise protect the brain from morphological and cognitive changes that occur following rTBI? Does timing of post-injury exercise initiation influence the recovery time course? We hypothesize that timing of post-injury running will influence recovery time. We predict that delayed post-injury exercise will be most beneficial in the cognitive and behavioral recovery.

## Materials and Methods

### Subjects

Adolescent male Sprague Dawley rats (100–140 g; 35 days old) were used in this study (Charles River, RRID:SCR_003792). This age group was chosen as the developmental profile is similar to the periadolescent human age group (30). Upon arrival rats were randomly assigned to injury (sham vs. rTBI) and running groups (locked or functional wheels pre- and post-injury). Animals were housed in pairs throughout entire study on a 12:12 light:dark cycle with food and water available *ad libutum*. All procedures were approved by The UCLA Chancellor's Animal Research Committee and National Institute of Health Guide for the Care and Use of Laboratory Animals and the Institutional Animal Care and Use Committee of Pepperdine University.

### Experiment 1: Development of the Rathlete Model

#### Exercise Training

Each evening for 10 consecutive days animals were placed individually into cages with either a functional (rathlete) or locked (sedentary) running wheel (Starr Life Sciences, Oakmont, PA) from 22:00 – 6:00 the next day. Animals were weighed each day upon removal from running wheel and placed back in homecage with their peer. These time periods were chosen to allow for exercise during their nocturnal cycle when they are most active, yet still allows for socialization, which is critical for adolescent development. The number of revolutions per minute was recorded for each rat using VitalView (VitalView Software, RRID:SCR_014497) v5.1 and analyzed across days and groups.

#### Rathlete Evaluation

**Figure 1:**
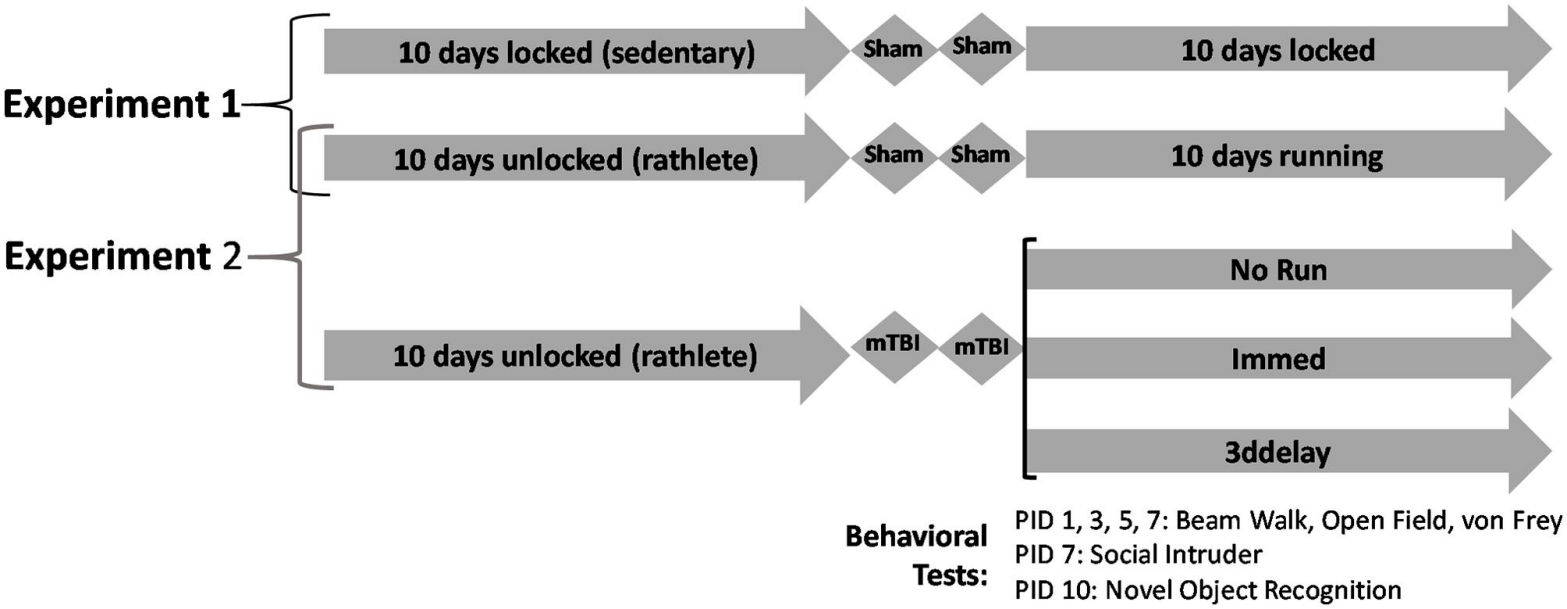
Experimental design. Experiment 1 analyzes differences between sedentary and exercised shams. Rats were given 10 days of voluntary access to locked or functional running wheels. Then they underwent 2 sham procedures, followed by another 10 days in their wheels. Experiment 2 analyzes effects of timing of return-to-activity following rTBI. Following 10 days in cages with locked or functional wheels, rats underwent 2 sham or mild closed head injuries, spaced 24 h apart. Following, rats were placed back in corresponding cages with locked or functional wheels. Behavioral tests were performed on post-injury days 1, 3, 5, 7, and 10.

To determine the benefits of exercise on brain, sedentary shams (*n* = 8) were compared to rathlete shams (*n* = 8). All underwent 2 sham procedures spaced 24 h apart, that included 2% isoflurane exposure until no longer responding to toe pinch. Rats were then returned to their homecage and allowed to recover. Each evening at 22:00, they were individually placed into cages until 6:00 the next morning with locked or functional wheels for another 10 consecutive days. Behavioral tests were conducted post-sham procedure (Figure 1).

### Experiment 2: Exercise Pre-conditioning on Recovery Following rTBI

To determine neural recovery profiles of those with prior exercise, sham rathletes were compared to rTBI rathletes. Rats were randomly assigned to groups upon arrival. Injured and sham rathletes were represented in all cohorts to ensure time was a confounding variable. Each night rats were individually placed in a cage with access to a functional running wheel for 10 days as described above. Following rTBI, rats were returned to their homecage. Each night rats were given access to running wheels according to the following groups: (A) Immediate access to a cage with a functional running wheel (rTBI+Immed, *n* = 6), (B) A cage with a locked wheel for 2 days followed by a functional wheel for 8 days (rTBI+3ddelay, *n* = 7), C) A cage with a locked wheel for 10 days (rTBI+norun, *n* = 7).

After the 2nd and final injury on days PID 1, 3, 5, 7 animals were tested in the following sequence for motor deficits (beam walking), anxiety-like (open field), and allodynia (Von Frey), described below. On PID 7 they were also tested for social interaction (social intruder) and on PID 10 for memory impairment (novel object recognition). Rats were euthanized on PID11 and tissue harvested for analysis (Figure 1).

#### Closed-Head Injury Model

The injury model used here was designed to reflect clinical aspects of a mTBI injury and has been described previously (31). This model causes no overt cell death, minimal axonal blebbing in ipsilateral white matter, and temporary behavioral impairment. Briefly, animals underwent 2% isoflurane anesthesia and were placed on a plexiglass block within a stereotaxic stage. The head was shaved and a mask was used to mark center of impact (AP: −3 mm, ML: −4 mm from Bregma). A metal piston (5 mm diameter, Hydraulic Control Inc., Emeryville, CA) was positioned on the skin's surface at 23° from the vertical and a controlled impact was delivered 8 mm at 36psi. The head was free to move in the direction of the injury. The injury results in brief apnea, delayed response to toe pinch, and increased righting times in the absence of mortality or skull fractures. In this study we aimed to determine consequences from rTBI. We have previously shown the metabolic dysfunction resulting from a single mTBI recovers by PID 3. When a second mTBI occurs prior to metabolic recovery, the metabolic dysfunction is cumulative and prolonged (31, 32). For this reason, we used a 24 h interval between 1st and 2nd injury in this study.

#### The Rat Standardized Concussion Assessment Tool (ratSCAT)

Our behavioral panel was designed to mimic the multimodal assessments performed in the clinical setting. Most post-concussive assessments are performed without any prior baseline exam, and instead are performed on multiple follow-up visits. The most widely used clinical test is the Sports Concussion Assessment Tool, a multipart tool which measures intensity of symptoms, cognition/memory, and balance.

In this report, the ratSCAT was created to align with clinical examinations. To assess motor and balance function, a beam walking task was performed; symptoms of anxiety were tested using an open field test where subjects are placed in a novel environment without ability to hide or escape; and headache/pain sensitivity tested using von Frey filaments to target neurological pain. Memory impairment and visual recognition was assessed using the novel object recognition (NOR) task. We also included a social interaction assessment using the social intruder (SI) test.

#### Beam Walking

This test was designed based on previous publications (33, 34). Animals were placed on wooden beam 35.5in long and 1 in. wide, 12in from the ground. At one end was a bright white light source, at the other an enclosed black safehouse box. A video camera was positioned at the end with the light facing the safe house, where animals were initially placed. On PID 1, 3, 5, 7, they were recorded walking toward safehouse and analyzed for time to cross beam (max 60 s), number of total right/left hindlimb steps, and number of left/right hindlimb slips. Animals were given 2 attempts per testing day. The first attempt was considered practice and may also contribute to learning the beam crossing task. To normalize this, only the 2nd attempt was analyzed for percentage of right or left hindlimb slips/total steps with hindlimbs and time to cross beam. Rats needing excessive encouragement to cross beam (>3 pushes and beam tapping) or not attempting to cross at all and were removed from analysis on that day of testing as they did not perform the task. These behaviors were represented in all groups and on all days tested. Statistical power was maintained across groups and test day. Expressed behavioral anomalies typically took place in both trials tested. A chi-square analysis did not indicate significant differences of non-performers between groups (X^2^ = 1.009, *p* = 0.91).

#### Open Field

On PID 1, 3, 5, 7, each rat was tested in the open field, which consisted of a white plywood box with a white plastic bottom measuring 27 × 27 × 15.” The outside walls were covered with black cardboard to prevent light penetration. On 3 of the sides were placed LED light strips to provide dim lighting evenly to the entire space. Each rat was placed in the center of the open field facing away from center and video-recorded from above for 5 min. These were later analyzed by individuals blind to animal condition. During analysis a clear screen was placed over the computer screen separating open field into 9 equally-sized squares. The following outcome measures were assessed: amount of time spent in center square, time spent in ambulation, time spent grooming, time spent immobilized, number of defecations and the number of rears.

Data transformation was performed using principal component analysis and scree plot to determine the lowest number of variables necessary to reliably represent a subject's anxiety-like behavior during the open field task. A factoral analysis was then run using a minimum residual or maximum likelihood function with an oblique rotation (“Promax”) using R (R Project for Statistical Computing, RRID:SCR_001905) 3.6.0. Following an initial analysis, variables with loading values <0.3 were removed and analyzed again. Results indicated that one factor was enough to describe 70% of the variability of the data. Variables were highly correlated with each other. As ambulation, rearing, and defecation may be more closely related to habituation in the open field arena, time spent in passive behavior (immobility, grooming, etc.) was analyzed between groups and test day. In rodent species, particularly anxious in open spaces, anxiolytic drugs decrease the passive behavior providing support that this behavior is more representative of anxiety-like behavior rather than habituation (35). The total time spent passive across all testing days was also calculated.

#### Von Frey

On PID 1, 3, 5, 7, neuropathic pain was testing using von Frey filaments. Animals were placed in an acrylic box (12.75 × 7.25 × 7”) with holes drilled in top for air circulation (amazon.com). They were allowed to acclimate for 10 min, while a filament was presented every 30 s. At times rats required more time than originally published to habituate to the test chamber. Sometimes, 30 min was allotted before testing with filaments. Following habituation, filaments were presented to the right (contralateral to injury) and left (Ipsilateral) periorbital space beginning with a force of 0.002 g and up to 6 g (Stoelting Co.). If threshold, see below, was achieved on the first filament, a lower threshold, 0.0008 was tested. Three consecutive stimulations were presented to each periorbital side, separated by 15 s intervals for each subject. Then the opposite side was tested for all animals, with right and left counterbalanced across filament size. False positives may occur during this test when utilized across multiple days. To avoid this, investigators were well-trained in the task. Further, to ensure threshold was met, more than 3 responses per hemisphere were recorded at times. Response to each stimulation was scored as follows: 0: no response, 1: Detection – head twitch or turn *toward* stimulation, 2: Withdrawal – head turn *away* from stimulus or paw swipe toward stimulus, 3: Escape/Attack – turns body away from stimulus or biting/attacking stimulus (36). For each hemisphere, 3 consecutive withdrawal responses (2+ rating) was considered threshold. If such a response was not observed by a filament force of 6 g, animals were given 6 g as their max threshold. On PID 1, 3, 5, 7 each side was analyzed for a) threshold obtained, b) total pain score (sum of scores up to and including threshold), c) percent response (trials with score ≥ 2/total trials).

#### Social Intruder

Beginning on PID 5, subjects were habituated to the task. The test was performed in a 10 g tank placed on a black surface with 3 of the 4 walls covered in black poster paper to reduce background lighting. All phases were performed under dim lights. A camera was positioned above tank as well as in front of uncovered side. Camera was placed prior to introducing any subject to arena. The test involved a habituation phase and testing phase. Habituation was performed over 2 days, during which each test subject and intruder was given individual access to arena for 7.5 min. Exploratory behavior was observed. During the testing phase, on PID7, individuals were isolated from their homecage for 1 h prior to testing. This was done to maximize social interaction behaviors. The test subject was placed in arena first, followed by intruder rat (marked on sides and top with black marker for identification). Intruder rats were slightly smaller and 1 week younger than test subjects to encourage interaction by test rat. Rats were analyzed for interaction with an intruder. Behaviors measured for test rat were: (a) time spent in play behavior (nape attacks, chasing, pinning), (b) time spent in social (sniffing/investigating) and general contact (crawling over, grooming intruder), (c) quantity play contacts, (d) quantity social contact, (e) quantity general contact, and (f) quantity avoidance (running away from intruder).

Data transformation with principal component analysis and screeplot indicated 2 components explained 96.5% of the variability in the data. A factor analysis was run with a minimum residual function including an oblique rotation (“Promax”) and 2 factors using R version 3.6.0. The results provided 2 main categories for analysis: play (loadings >0.8 for MR1) and contact (loadings >0.6 for MR2). NOTE: intruder rats did not arrive on time to test sedentary shams. This group was not included in analysis.

#### Novel Object Recognition

This test has been previously described (37). Briefly, subjects were given a habituation period which allowed for 10 min of exploration in an open arena (same as open field). The testing phase followed, consisting of a familiarization training trial followed by a novel test trial on PD10. In the familiarization trial, 2 identical objects were placed near the top of the arena and animals were placed on the opposite end with backs to the objects to prevent coercion of object interaction. Over 5 min, subjects could freely explore arena as well as objects. The novel trial was performed 24 h later as this time has shown to have the most robust object recognition in this age group (38). Additionally, this interval has shown greatest impairment following a single mTBI and rTBI (31). In the novel trial, one object was replaced by a new object (right and left randomly assigned), and subjects were once again allowed 5 min to explore. For both familiar and novel trials, subjects were analyzed for (A) number of object contacts and (B) time spent with each object. The difference in time spent with novel object was compared to corresponding object of previous day. Additional factors observed were object of first contact, exploration, rearing, time grooming, time immobile, and defecations.

#### End Point and Western Blot Analysis

After the last day of post-injury running (PID11) animals were euthanized and brains were retrieved for analysis. Subjects were briefly anesthetized under isoflurane (3%) until no longer responding to toe pinch when they underwent rapid decapitation. Brains were carefully removed and dissected on ice. Right and left brain regions were separated. Parietal cortex and hippocampus were isolated from each hemisphere and stored at −80°C until analysis. Additionally, the gastrocnemius muscle was dissected and stored for analysis. A Western blot was run to measure levels of brain derived neurotrophic factor (BDNF) in the cortex and hippocampus, along with peroxisome proliferator-activated receptor gamma coactivator 1-α (PGC1α, marker of mitochondrial biogenesis) levels in the cortex, hippocampus, and muscle. For BDNF, samples were homogenized in radioimmunoprecipitation assay (RIPA) buffer (Thermo-Fisher, Waltham, MA) and were centrifuged at 30,000 g for 20 min at 4°C. PGC1α, samples underwent nuclear extraction (NE-PER, Thermo-Fisher) with centrifugation at 30,000 g for 10 min at 4°C. The supernatant was removed and a protein assay was performed using a BCA kit. Protein (10 μg) samples were separated on a 4–12% polyacrylamide gel (Bio-Rad Laboratories, Hercules, CA) for all tests. Separated proteins were then transferred to a polyvinylidene difluoride (PVDF) membrane. SYPRO Ruby Protein Blot Stain (Thermo-Fisher) was first run to visualize the efficiency of the protein transfer and if lanes were loaded equally. Total protein transferred was used to normalize quantity of BDNF and PGC1α protein. Following Ruby analysis, membrane was blocked for 1 h using 5% skim milk in TBST (TBS containing 0.1% Tween20), then incubated overnight at 4°C in primary antibody (anti-BDNF, 1:5000, Abcam Cat# ab108319, RRID:AB_10862052, anti-PGC1α, 1:5000, Abcam Cat# ab54481, RRID:AB_881987, Cambridge, MA). Samples were washed in TBST, blocked with 5% skim milk in TBST and then incubated for 1 h in secondary antibody (anti-rabbit, 1:10000, Vector Laboratories Cat# BA-1000, RRID:AB_2313606) at room temperature. Immunoreactivity was detected using ECLplus kits (Thermo-Fisher). BDNF band was read at 17 kD and PGC1α was read at 102 kD.

#### Statistics

Power analyses were performed prior to study by statistician, Dr. McArthur to determine sufficient n per group using G^*^Power v.3.1.9.4. A sample size of *n* = 8/group for experiment 1 and *n* = 6/group for experiment 2 was found to be sufficient for statistical power at the recommended 0.80 effect size and α error of 0.05 level. Subjects were randomly assigned to groups at start of experiments, and analyzed blind to condition. Data are presented as average ± SEM. Where appropriate, holm-corrected *t*-tests were performed to compare sedentary to rathlete shams. A linear regression was also performed on foot slips to determine learning of task. Additionally, in the von Frey test, left (ipsilateral) and right (contralateral) periorbital responses were compared using a paired *t*-test for each rat. For memory task, a repeated measures two-way ANOVA with holm-corrected *t*-test compared percentage of time spent with novel object on novel (test) day to familiar (train) day.

To compare rathlete shams and rTBI groups, body weight gain (pre- and post-rTBI) was analyzed using a one-way ANOVA. Running distance was compared using a repeated-measures ANOVA, with holm-corrected *t*-test. A mixed-model ANOVA with test day being the repeated measure was performed on behavioral assessments. A one-way ANOVA was also performed the open field (total passive behavior), social intruder test, and protein expression from Western blot. Holm-corrected *post-hoc t*-tests were performed with statistical significance (α =0.05). Data were analyzed using R Program v 3.6.0 and GraphPad Prism (GraphPad Prism, RRID:SCR_002798) version 8.4.3.

## Results

### Experiment 1: Development of the Rathlete Model

#### Rathlete Evaluation

Rathlete shams (*n* = 8) were given 10 days of voluntary access to a running wheel. Rats began running on the first day with an average distance of 1271.5 ± 178.6 m and intensity of 6.7 ± 0.6 m/min. Distance run and intensity gradually increased each day of running until day 5 when they plateaued at 3146.1 ± 923.8 m/day and 17.6 ± 3.3 m/min.

#### Body Weight

Average weight gain of sedentary shams (*n* = 8) was 80.0 ± 3.14 g pre-sham procedure and 76.6 ± 4.24 g post-procedure. Rathlete shams showed average gain of 73.0 ± 2.90 g pre-injury and 67.5 ± 3.38 g post-injury. Weight difference did not differ between sedentary and rathlete shams [Student's *t*-test, pre: *t*_(14)_ = 1.63, *p* = 0.12, post: *t*_(4)_ = 1.68, *p* = 0.11] (data not shown).

#### Motor Skills

**Figure 2:**
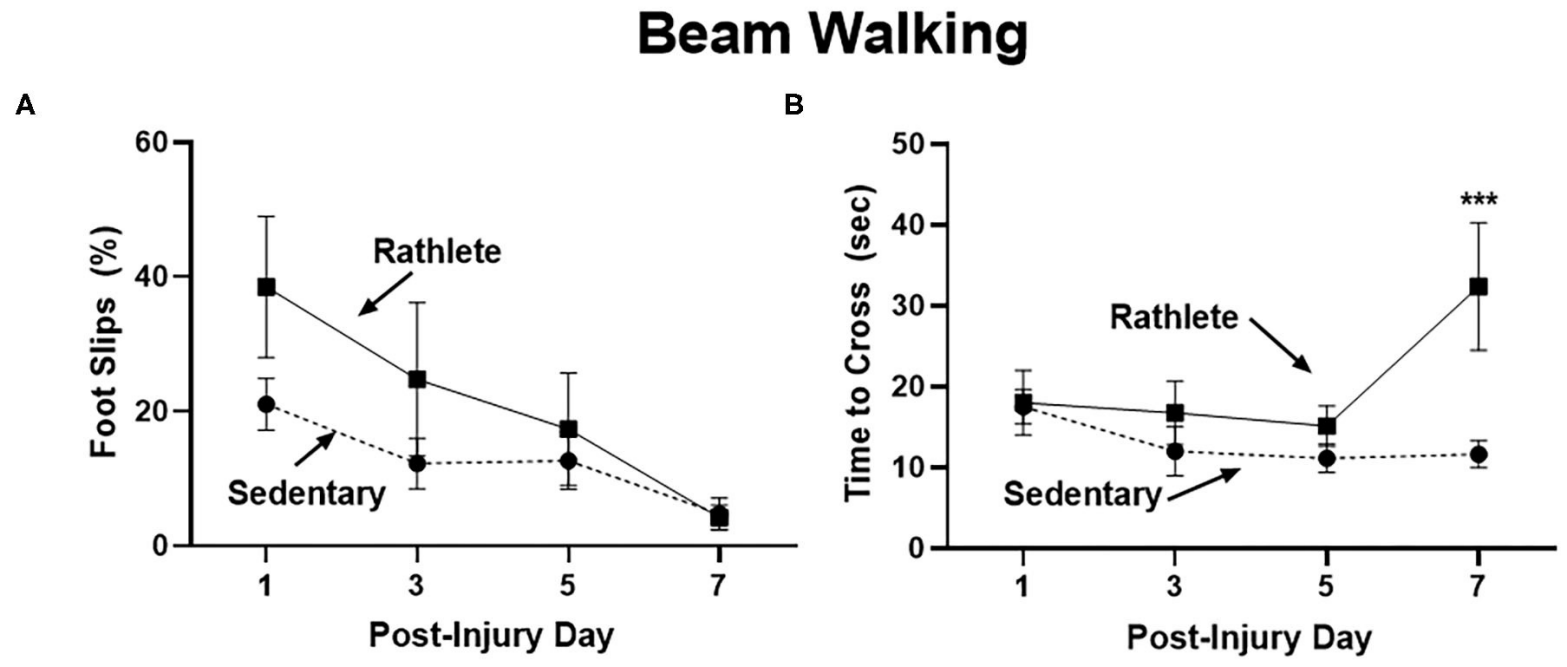
Beam walking task for motor impairment. Adolescent rathlete shams (solid line) were given 10 days of voluntary access to a running wheel. Sedentary shams (dashed line) were given 10 days in a locked running wheel. Motor coordination was analyzed using a beam walking task following sham procedure on PID 1, 3, 5, and 7. Proportion of hindlimb slips **(A)** and time to cross beam **(B)** were calculated and compared between groups. ***p < 0.001.

A beam walk task was performed as a measure of fine motor skills and balance impairment (Figure 2). Proportion of steps that resulted in a hindlimb slip were calculated for the right and left side, as well as time to cross beam. No significant differences were seen with number of right and left slips, so data were combined. Results showed that sedentary shams performed similarly crossing the beam compared to rathlete shams on all days tested [*t*'s_(13)_ = 0.18–1.37, *p* = 0.58–0.87] (Figure 2A). A linear regression was performed to test the change in performance on the beam walk task across the multiple days of testing. Both rathlete and sedentary shams showed significant improvement in the motor task over the days of testing (r^2^ = 0.42–0.56, *p*'s = 0.006–0.007). No differences were observed in time to cross beam on post-injury days 1, 3, or 5 (Figure 2B). Rathlete shams were significantly slower (37 ± 6.84 s) than sedentary shams (12.57 ± 1.53 s) on PID7 [holm-corrected *t*-test: *t*_(52)_ = 4.03, *p* < 0.001], when the majority of rathletes (5/8) took longer to cross beam.

#### Anxiety

**Figure 3:**
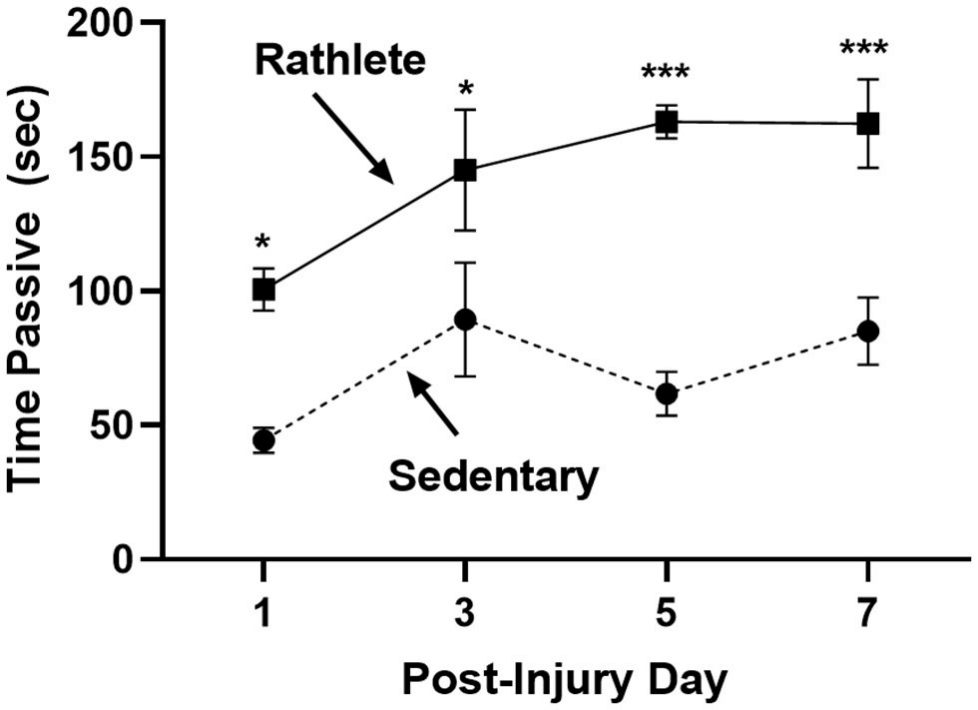
Open field test for anxiety: Rathlete shams (solid line) were compared to sedentary shams (dashed line) for performance in an open field task. Following repeat sham procedure, rats were tested for time spent immobile over the 5 min of the task. Rathlete shams showed more time in passive (immobile) time. *p < 0.05, ***p < 0.001.

The open field was performed to determine stress-induced anxiety-like behavior for rats, where time spent in passive behavior relates to high anxiety (Figure 3). Rathlete shams consistently spent more time immobile over all the days tested [*t*'s_(55)_ = 2.71–5.06, *p*'s = 0.01–0.001]. This indicates that voluntary running sensitizes rats to an open field task. Other behaviors tested included total time active, time spent in center square, time spent grooming, number of defecations, and number of rears (Table 1). Only time spent immobile was significant between groups.

**Table 1:** Behaviors of anxiety in open field test.

		**Exploratory**		**Immobility**		**Habituation**	
		**Time active**	**Time in center**	**Time passive**	**Time grooming**	**Defecation**	**Rearing**
Day 1	Sedentary sham	255.75 (4.24)	28.50 (5.10)	44.25 (4.24)^*^	23.12 (3.96)	0.62 (0.37)	35.27 (2.72)
	Rathlete sham	190.25 (9.00)	2.62 (1.66)	100.62 (7.95)	15.37 (1.72)	0 (0)	24.75 (1.56)
	rTBI no run	201.30 (12.20)	4.70 (1.10)	68.40 (13.4)	23.30 (5.80)	2.0 (0.70)	22.00 (1.60)
	rTBI immed	198.70 (8.50)	4.30 (1.70)	80.80 (13.80)	22.70 (4.50)	1.30 (1.00)	27.80 (2.50)
	rTBI 3ddelay	211.70 (14.40)	9.30 (1.20)	53.60 (9.90)	31.30 (10.30)	1.60 (0.80)	34.10 (2.90)
Day 3	Sedentary sham	225.80 (9.90)	14.80 (2.70)	86.80 (19.70)^*^	30.60 (7.90)	0.50 (0.40)	33.40 (2.30)
	Rathlete sham	133.75 (26.44)	2.25 (1.10)	145.12 (22.60)	19.87 (2.22)	0.75 (0.37)	16.50 (2.97)
	rTBI no run	133.90 (23.10)	3.40 (1.50)	138.70 (24.7)	24.60 (7.80)	1.30 (0.60)	13.90 (3.70)
	rTBI immed	146.00 (21.90)	4.50 (2.30)	116.50 (16.00)	34.00 (7.80)	1.80 (1.20)	19.20 (4.50)
	rTBI 3ddelay	172.90 (22.00)	5.90 (1.90)	99.60 (20.10)	29.30 (4.80)	0.60 (0.40)	22.10 (2.70)
Day 5	Sedentary sham	241.12 (7.96)	15.00 (3.55)	59.00 (8.04)^***^	29.75 (10.55)	1.62 (1.10)	32.50 (2.78)
	Rathlete sham	145.62 (11.08)	1.87 (0.63)	163.00 (6.26)	14.87 (3.11)	0 (0)	20.75 (1.33)
	rTBI no run	81.10 (26.60)	1.00 (0.80)	193.90 (28.90)	24.70 (9.60)	0.40 (0.40)	11.60 (3.50)
	rTBI immed	136.00 (32.50)	10.20 (6.30)	133.50 (34.10)	34.50 (11.20)	1.00 (0.60)	17.50 (5.30)
	rTBI 3ddelay	150.40 (13.10)	3.00 (1.10)	111.40 (15.20)	40.70 (11.90)	0.60 (0.40)	22.10 (2.50)
Day 7	Sedentary sham	226.50 (19.40)	16.00 (3.00)	86.10 (11.70)^***^	17.00 (3.90)	0.30 (0.30)	31.50 (2.70)
	Rathlete sham	116.12 (18.81)	1.37 (1.37)	162.37 (16.60)	16.12 (2.72)	0 (0)	16.12 (1.57)
	rTBI no run	103.30 (23.50)	1.30 (0.70)	159.70 (25.40)	32.70 (12.50)	0 (0)	13.10 (3.80)
	rTBI immed	163.50 (29.00)	9.30 (6.80)	110.50 (28.80)	25.80 (5.70)	0.80 (0.80)	16.00 (3.00)
	rTBI 3ddelay	125.70 (20.30)	5.10 (1.80)	141.70 (22.10)	29.30 (5.10)	0.40 (0.40)	16.10 (2.60)

*Total time spent over 5min (300sec) of test was calculated for behaviors related to exploration, immobility, and habituation. Bold font indicated significance. Sedentary vs. Rathlete sham*.

* *p < 0.05*,

*** *p < 0.001*.

#### Allodynia

**Figure 4:**
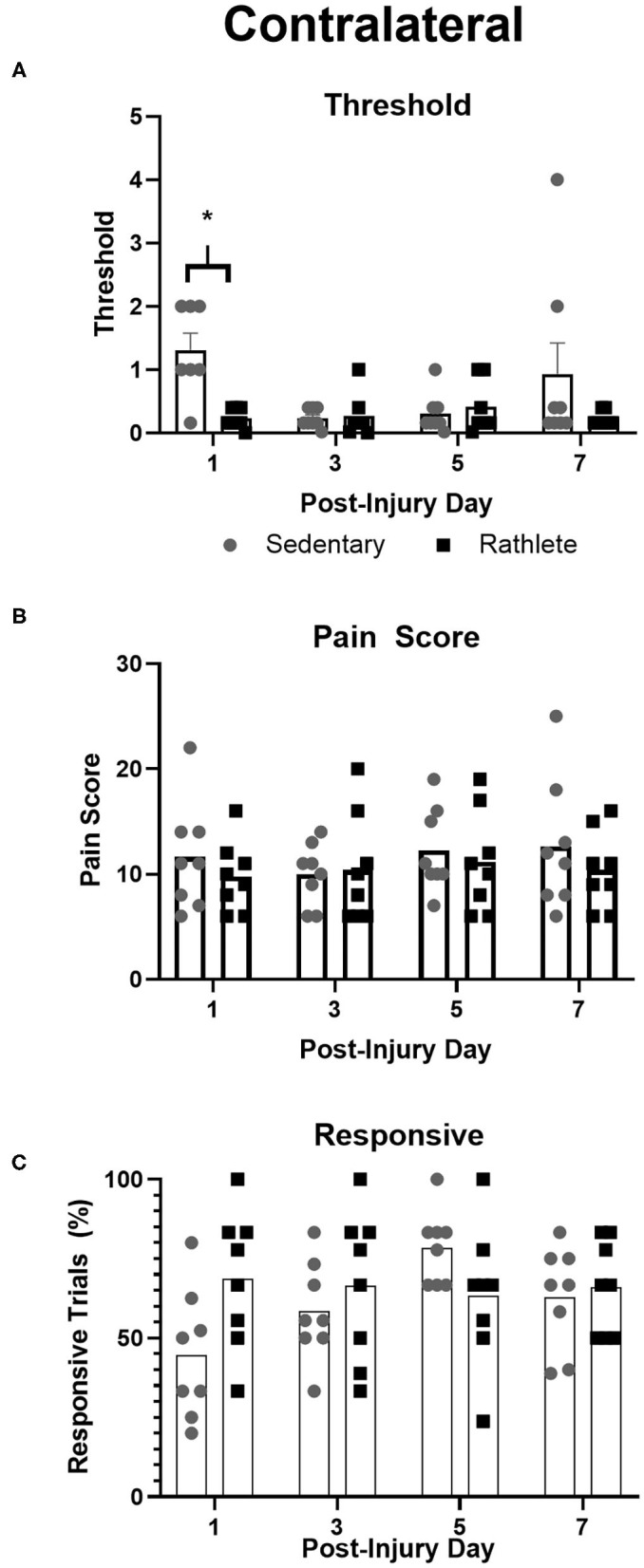
Von Frey Test of allodynia. Neuropathic pain, a marker for “headache,” was measured in rathlete and sedentary shams using von Frey filaments. Threshold **(A)** indicated the smallest size filament that caused a withdraw reaction 3 consecutive times. Pain score **(B)** was the sum of responses up to and including threshold. Responsive **(C)** was the percentage of withdraw responses. *p < 0.05.

To test for neuropathic pain, von Frey filaments were used to determine pain threshold, pain score, and pain responsiveness for right and left periorbital hemispheres. All rats showed high activity following the 10 min habituation period. As such, testing with filaments was performed while subject was rearing, which was not ideal and may have resulted in false positive responses. Attempts were made to confirm response in such cases, but may still be present as confounding variable in the analysis. Some subjects responded strongly to filament presentation. For a result of no response, behavior was clear. This type of response was not consistent across days for any one animal and as such there were no omitted data points or outliers. Sedentary shams showed similar right and left scores to periorbital pain threshold [*t*_(31)_ = 1.25, *p* = 0.22], pain response [*t*_(31)_ = 0.02, *p* = 0.98] and responsivity [*t*_(31)_ = 1.12, *p* = 0.27]. Rathlete shams, on the other hand, displayed greater responsivity on the right periorbital hemisphere compared to left [overall: *t*_(31)_ = 4.63, *p* < 0.0001, day 1: *t*_(7)_ = 2.99, *p* = 0.02, day 3: *t*_(7)_ = 1.56, *p* = 0.16, day 5: *t*_(7)_ = 1.97, *p* = 0.09, day 7: *t*_(7)_ = 3.31, *p* = 0.01]. Right and left sides showed similar results for periorbital threshold [*t*_(31)_ = 0.68, *p* = 0.50] and pain score [*t*_(31)_ = 0.50, *p* = 0.62]. Mean threshold was similar for sedentary and rathlete shams on all days tested except for PID 1 (Figure 4A). Sedentary shams showed significantly higher response threshold on PID 1 compared to rathlete shams [average 1.89 vs. 0.23, holm-corrected *t*-test *t*_(56)_ = 2.82, *p* = 0.03]. No differences were seen on PID 3 [0.23 vs. 0.74, *t*_(56)_ = 0.86, *p* = 0.45], day 5 [0.31 vs. 1.11, *t*_(56)_ = 1.36, *p* = 0.45], or PID 7 [0.93 vs. 0.22, *t*_(56)_ = 1.20, *p* = 0.45]. Mean pain scores remained consistent at all timepoints for both groups (Figure 4B). No significant differences were seen for response sensitivity, nor overall responsiveness to the task (Figure 4C).

#### Memory Impairment

NOR is a test of working memory. Following a 24 h interval, rats are able to recognize and spend >50% time exploring a novel object (31, 37). Both sham groups increased time spent with novel object compared to 24 hr earlier (*F*_(1, 14)_ = 13.0, *p* = 0.003; rathlete: *t*_(14)_ = 3.02, *p* = 0.02, sedentary: *t*_(14)_ = 2.64, *p* = 0.04). Time spent with novel object was similar in sedentary 61 ± 1.84% and rathlete 69 ± 3.33% shams, and results did not differ *F*_(1, 14)_ = 1.48, *p* = 0.23. There were also no differences in overall behavior in the arena. During the familiarization phase, there was no difference in the total amount of time spent with both objects [sedentary sham: 40 ± 6 s, rathlete sham = 51 ± 6 s).

#### Protein Expression

**Figure 5:**
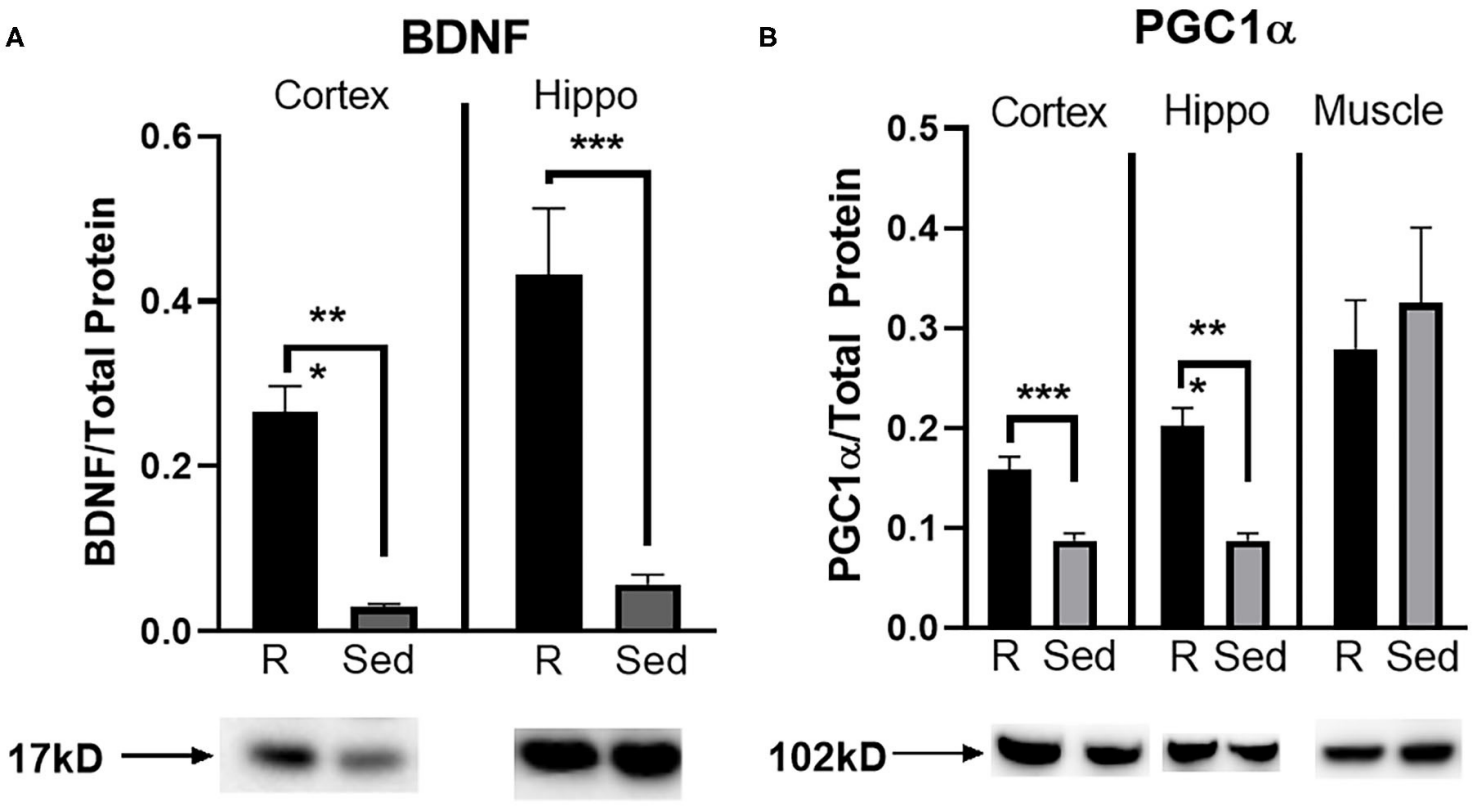
Protein expression in tissue homogenate. Parietal lobe (cortex) and hippocampal (hippo) brain tissue, and gastrocnemius (muscle) tissue was isolated from rathlete (black) and sedentary (gray) adolescent shams. Following homogenization, cells were isolated and analyzed using Western blot for **(A)** brain-derived neurotrophic factor (BDNF), a marker of neuroplasticity, and **(B)** peroxisome proliferator-activated gamma coactivator 1-alpha (PGC1α), a regulator of mitochondrial biogenesis and function. Density of BDNF and PGC1α protein was normalized using Ruby and presented as proportion of protein in tissue. *p < 0.05, **p < 0.01, ***p < 0.001.

A Western blot was run to determine protein concentration of BDNF and PGC1α in the parietal cortex, hippocampus, and muscle (PGC1α only). Rathlete shams showed significantly greater BDNF expression in both the cortex [*t*_(28)_ = 3.84, *p* < 0.001] and hippocampus [*t*_(28)_ = 6.07, *p* < 0.001] (Figure 5A). Expression of PGC1α was also greater in rathlete shams in the parietal cortex [*t*_(14)_ = 4.86, *p* < 0.001], and hippocampus [*t*_(14)_ = 5.64, *p* < 0.001], but not the muscle [*t*_(19)_ = 0.44, *p* = 0.66] (Figure 5B).

### Experiment 2: Exercise Pre-conditioning on Recovery Following rTBI

#### Body Weight

**Figure 6:**
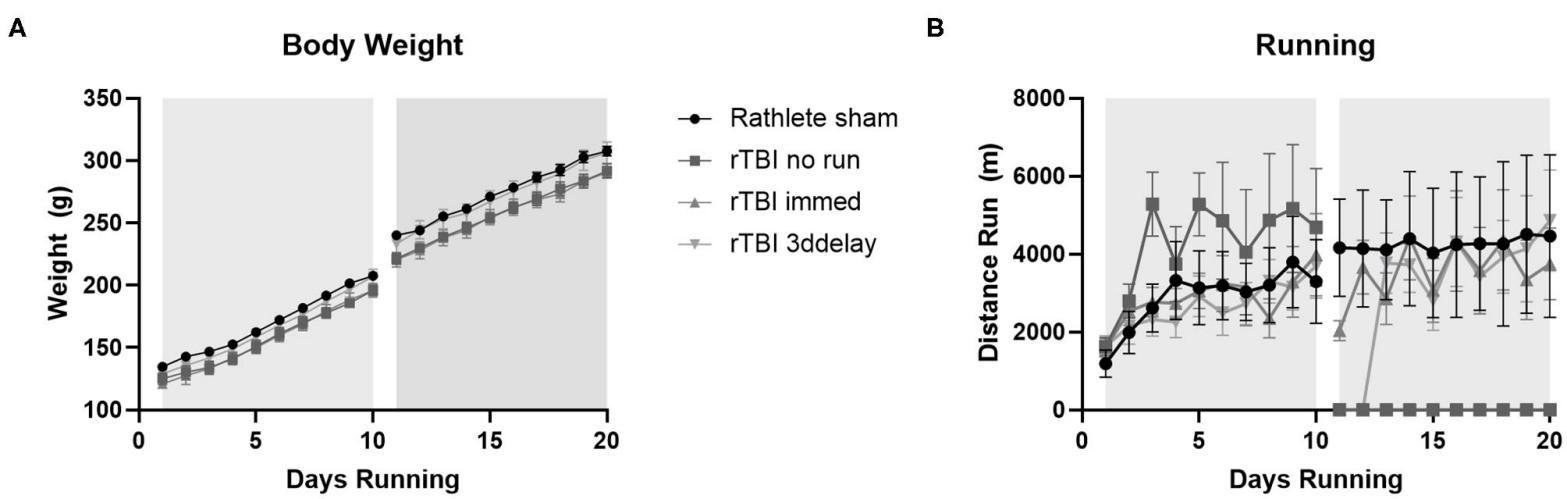
Body weight changes. All rats received voluntary access to a running wheel for 10 days, 8 hr/day, before repeat sham (rathlete) or closed head injury (rTBI). Following, one group was given another 10 days of voluntary running (rTBI+immed), another was given 3 days in a locked wheel before open access (rTBI+3ddelay), and a last group was given 10 days in a locked wheel (rTBI+no run). **(A)** Weights were taken at 6 a.m., upon removal from wheels. **(B)** Distance run was computer-monitored each day.

Pre-injury weights for all groups ranged 121–134 g and ending weights ranged 290–307 g (Figure 6A). Average weight gain pre-injury for all groups ranged from 71 to 78 g, and post-injury was 67–73 g. There were no significant differences in weight gain between any of the groups [one-way ANOVA pre-injury *F*_(3, 27)_ = 0.84, *p* = 0.49 and post-injury, *F*_(3, 27)_ = 0.45, *p* = 0.71].

#### Running

Voluntary running occurred each evening and increased until injury (Figure 6B). A repeated measure ANOVA found significant pre-injury difference for group [*F*_(3, 39)_ = 21.91, *p* < 0.001]. Daily pre-injury running distance was similar to rathlete shams from Experiment 1 (2,877 ± 241 m/day), rTBI+Immed (2,872 ± 2036 m/day), and rTBI+3ddelay (2,667 ± 195 m/day), though pre-injury running distance of rTBI+no run (4,237 ± 383) was significantly greater compared to all other groups [*t*'s_(9)_ = 4.7–6.0, *p*'s = 0.002–0.004] (Figure 6B). Post-injury running distance also differed between groups [*F*_(3, 39)_ = 47.90, *p* < 0.001]. Running wheel distance was greatest in sham rathletes, followed by rTBI+Immed, then rTBI+3ddelay. Rathlete shams ran significantly further than rTBI+Immed group [*t*_(9)_ = 3.21, *p* = 0.02].

#### Motor Skills

**Figure 7:**
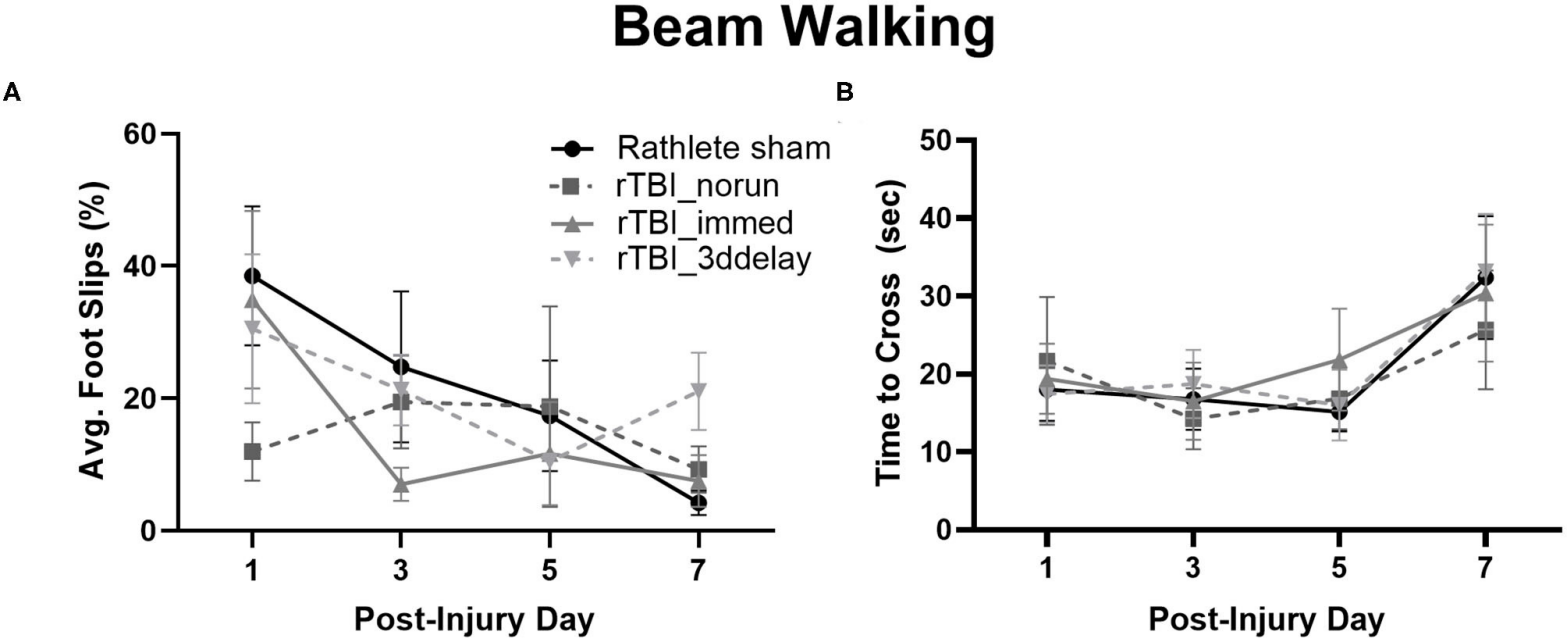
Beam Walking of Rathletes. Adolescent rathletes were given 10 days of voluntary access to a locked or functional running wheel. Motor coordination was analyzed using a beam walking task following sham or rTBI on PID 1, 3, 5, and 7. Proportion of hindlimb slips **(A)** and time to cross beam **(B)** were calculated and compared between groups.

Assessment of motor function following rTBI was tested in rathletes (Figure 7). Hind limb slips were measured on PID 1, 3, 5, and 7. A repeated measures ANOVA did not find differences between groups [*F*_(3, 15)_ = 0.64, *p* = 0.56], but there was trend for day [*F*_(3, 15)_ = 3.4, *p* = 0.07] (Figure 7A). A linear regression indicated that none of the injured rathletes showed a significant improvement in the beam walking task over the 7 days following injury (*r*^2^ = 0.02-.04, ps = 0.07–0.64), unlike rathlete shams. There was also no correlation with foot faults to distance run (*r*^2^s = 0–0.60, *p* = 0.12–0.99). This indicates that voluntary exercise or fatigue was not a factor in the motor task.

Lastly, time to cross the beam was analyzed to determine if it may be a covariate in the foot fault and scoring analysis (Figure 7B). No significant differences were seen for any group [*F*_(3, 15)_ = 0.56, *p* = 0.56], though there was for test day [*F*_(3, 15)_ = 21.71, *p* < 0.001]. Linear regressions were not significant (*r*^2^s = 0–0.11, ps = 0.09–0.96). This indicates that lengthened time did not significantly improve beam walking score.

#### Anxiety-Like Behavior

**Figure 8:**
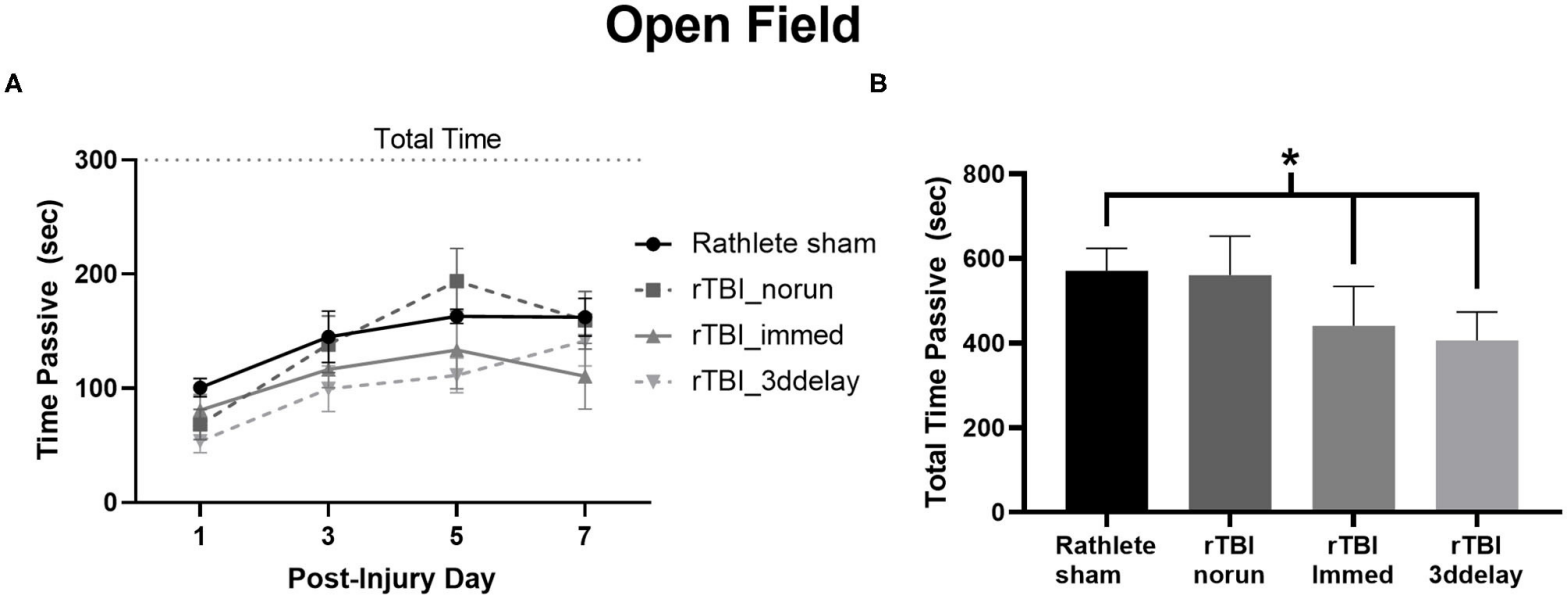
Measuring anxiety-like behavior in Rathletes. Following repeat sham or rTBI, rats were placed in an open field arena and tested for time spent immobile (passive) over the 5 min of the task **(A)**. The sum of immobility (passive) time over all 4 testing days was calculated and compared across groups **(B)**. *p < 0.05.

An open field test was performed to observe exploratory and passive behavior on PID 1, 3, 5, and 7 (Figure 8A) and total time across all 4 testing days (Figure 8B). A repeated-measures ANOVA showed a significant effect of time [*F*_(3, 72)_ = 15.90, *p* < 0.001], but not for group [*F*_(3, 24)_ = 2.33, *p* = 0.10]. Time spent immobile significantly increased over testing days [*t*s_(27)_ = 5.94–8.37, *p*s < 0.001]. Summing across all testing days, rathlete shams showed higher anxiety-like behaviors than rTBI+Immed [holm-corrected *t*_(3)_ = 4.75, *p* = 0.04] and rTBI+3ddelay [*t*_(3)_ = 5.91, *p* = 0.03] (Figure 8B).

#### Allodynia

Changes in neuropathic pain perception in rathletes as a result of rTBI was analyzed using von Frey filaments (Table 2). Rats in rTBI+no run group showed greater responsivity in the contralateral (right) vs. ipsilateral (left) hemisphere [overall: *t*_(27)_ = 3.982, *p* < 0.001, day 1: *t*_(6)_ = 2.98, *p* = 0.02, day 3: *t*_(27)_ = 1.56, *p* = 0.17, day 5: *t*_(6)_ = 0.88, *p* = 0.41, day 7: *t*_(6)_ = 2.22, *p* = 0.07]. Pain scores and periorbital threshold showed a trend for hemispheric differences, but were not significant [*t*_(27)_ = 1.33, *p* = 0.19, *t*_(27)_ = 1.59, *p* = 0.12, respectively]. Rats in rTBI+3dd group did not show hemispheric differences to pain score [*t*_(27)_ = 1.77, *p* = 0.09], threshold [*t*_(27)_ = 1.66, *p* = 0.10], and responsivity [overall: *t*_(27)_ = 1.35, *p* = 0.19, day 1: *t*_(6)_ = 2.56, *p* < 0.05]. Nor did rats in rTBI+Immed group to pain score [*t*(23) = 0.19, *p* = 0.84], periorbital threshold [*t*_(23)_ = 0.40, *p* = 0.69]. They did show greater responsivity on the contralateral side [overall: *t*_(23)_ = 2.55, *p* = 0.02, day 1: *p* = 0.18, day 3: *t*_(5)_ = 0.75, *p* = 0.49, day 5: *t*_(5)_ = 0.69, *p* = 0.52, day 7: *t*_(5)_ = 2.27, *p* = 0.07]. Group analyses were performed on contralateral and ipsilateral sides separately.

**Table 2:** Response to von Frey Filaments.

		**Threshold**		**Pain Score**		**Responsive (%)**	
		**Ipsi**	**Contra**	**Ipsi**	**Contra**	**Ipsi**	**Contra**
Day 1	Rathlete sham	0.53 (0.23)	0.23 (0.05)	9.50 (0.93)	9.75 (1.18)	50.06 (5.27)	**68.75 (7.64)#**
	rTBI no run	1.53 (0.78)	1.04 (0.83)	11.57 (1.67)	9.86 (0.67)	48.51 (5.82)	**69.27 (9.74)#**
	rTBI immed	0.21 (0.06)	0.25 (0.15)	8.00 (0.89)	9.17 (1.28)	61.11 (7.17)	71.30 (6.79)
	rTBI 3ddelay	1.39 (0.78)	0.19 (0.06)	10.14 (1.67)	8.71 (1.39)	45.07 (5.81)	**73.81 (8.37)#**
Day 3	Rathlete sham	0.47 (0.16)	0.74 (0.48)	10.25 (1.39)	10.37 (1.83)	53.12 (4.44)	66.67 (8.40)
	rTBI no run	1.04 (0.83)	1.00 (0.84)	8.43 (1.02)	9.57 (1.80)	47.96 (7.53)	59.52 (9.52)
	rTBI immed	0.25 (0.15)	0.23 (0.08)	8.67 (0.99)	8.33 (1.56)	62.50 (9.56)	66.20 (11.26)
	rTBI 3ddelay	0.56 (0.27)	0.20 (0.07)	12.86 (1.28)	8.29 (0.89)	71.83 (9.24)	71.43 (9.34)
Day 5	Rathlete sham	1.28 (0.71)	1.11 (0.71)	11.00 (1.32)	11.12 (1.69)	50.68 (5.48)	65.48 (9.31)
	rTBI no run	1.07 (0.83)	1.00 (0.84)	7.57 (1.70)	11.86 (1.22)	58.33 (13.24)	65.42 (9.47)
	rTBI immed	0.15 (0.06)	0.29 (0.15)	8.50 (0.88)	9.17 (1.76)	66.67 (6.73)	75.00 (8.33)
	rTBI 3ddelay	0.99 (0.84)	1.00 (0.84)	9.29 (1.46)	9.29 (1.27)	55.44 (10.24)	64.85 (12.22)
Day 7	Rathlete sham	0.26 (0.05)	0.22 (0.04)	8.87 (1.16)	10.37 (1.31)	52.08 (5.55)	**65.97 (5.19)#**
	rTBI no run	0.19 (0.06)	0.31 (0.13)	9.29 (1.13)	11.57 (1.88)	55.56 (4.70)	76.98 (8.78)
	rTBI immed	0.40 (0.14)	0.19 (0.07)	8.50 (1.15)	8.17 (1.01)	52.31 (10.20)	74.07 (9.37)
	rTBI 3ddelay	0.37 (0.28)	0.37 (0.28)	9.57 (1.69)	9.86 (1.87)	83.81 (7.81)^*^	76.03 (7.60)

*Filaments of different sizes ranging from 0.008 g to 6 g were used to measure neuropathic pain, or “headache.” Threshold is the largest filament size that elicited 3 consecutive withdrawal responses from rats. Pain Score indicated sum of all responses up to and including threshold. Responsive indicated proportion of responses ≥2 compared to all responses. Bold font indicates significance. Ipsi, ipsilateral; contra, contralateral. Compared to rathlete sham*,

* *p < 0.05, Ipsi vs. Contra*,

# *p < 0.05*.

#### Contralateral

Overall, all rathletes showed similar results for pain threshold, pain score, and responsiveness to von Frey filaments (Table 2). There was a significant group difference to pain score [*F*_(3, 15)_-10.01, *p* = 0.02), with rTBI+Immed showing significantly lower pain score than rathlete shams [Holm-corrected *t*-test, *t*_(3)_ = 4.53, *p* = 0.04]. Test day was not a significant measure [*F*_(3, 15)_ = 3.18, *p* = 0.08]. Pain threshold also showed a trend, but a repeated measures one-way ANOVA did not indicate significance [group: *F*_(3, 15)_ = 3.42, *p* = 0.10, time: *F*_(3, 15)_ = 3.25, *p* = 0.07]. Responsiveness was not significant [group: *F*_(3, 15)_ =1.70, *p* = 0.26, time: *F*_(3, 15)_ = 2.56, *p* = 0.11]. Subject variability was significant, accounting for 46% of the variation in pain threshold [*F*_(24, 72)_ = 3.38, *p* < 0.001] and 49% for responsiveness [*F*_(24, 72)_ = 3.18, *p* < 0.001] data, but not pain score [20%, *F*_(24, 72)_ = 0.85, *p* = 0.66].

#### Ipsilateral

A two-way repeated measures ANOVA showed significant interaction difference in responsiveness to von Frey filaments [Group: *F*_(3, 24)_ = 1.38, *p* = 0.27; Time: *F*_(3, 72)_ = 1.74, *p* = 0.17; Interaction: *F*_(9, 72)_ = 2.32, *p* = 0.02]. A holm-corrected multiple comparisons test indicated that rTBI+3ddelay had higher responsive values compared to rathlete shams on day 7 of testing [*t*_(9)_ = 11.15, *p* = 0.02]. No differences were observed for pain threshold [Group: *F*_(3, 24)_ = 0.52, *p* = 0.67; Time: *F*_(3, 72)_ = 2.38, *p* = 0.09; Interaction: *F*_(9, 72)_ = 0.98, *p* = 0.47] nor pain score [Group: *F*_(3, 24)_ = 1.03, *p* = 0.32; Time: *F*_(3, 72)_ = 0.72, *p* = 0.52; Interaction: *F*_(9, 72)_ = 1.35, *p* = 0.23]. Subject variability was significant and accounted for 34% of the variation for pain score (*p* = 0.01), 55% to pain threshold (*p* < 0.0001), and 38% to responsiveness (*p* < 0.001).

#### Social Interaction

**Figure 9:**
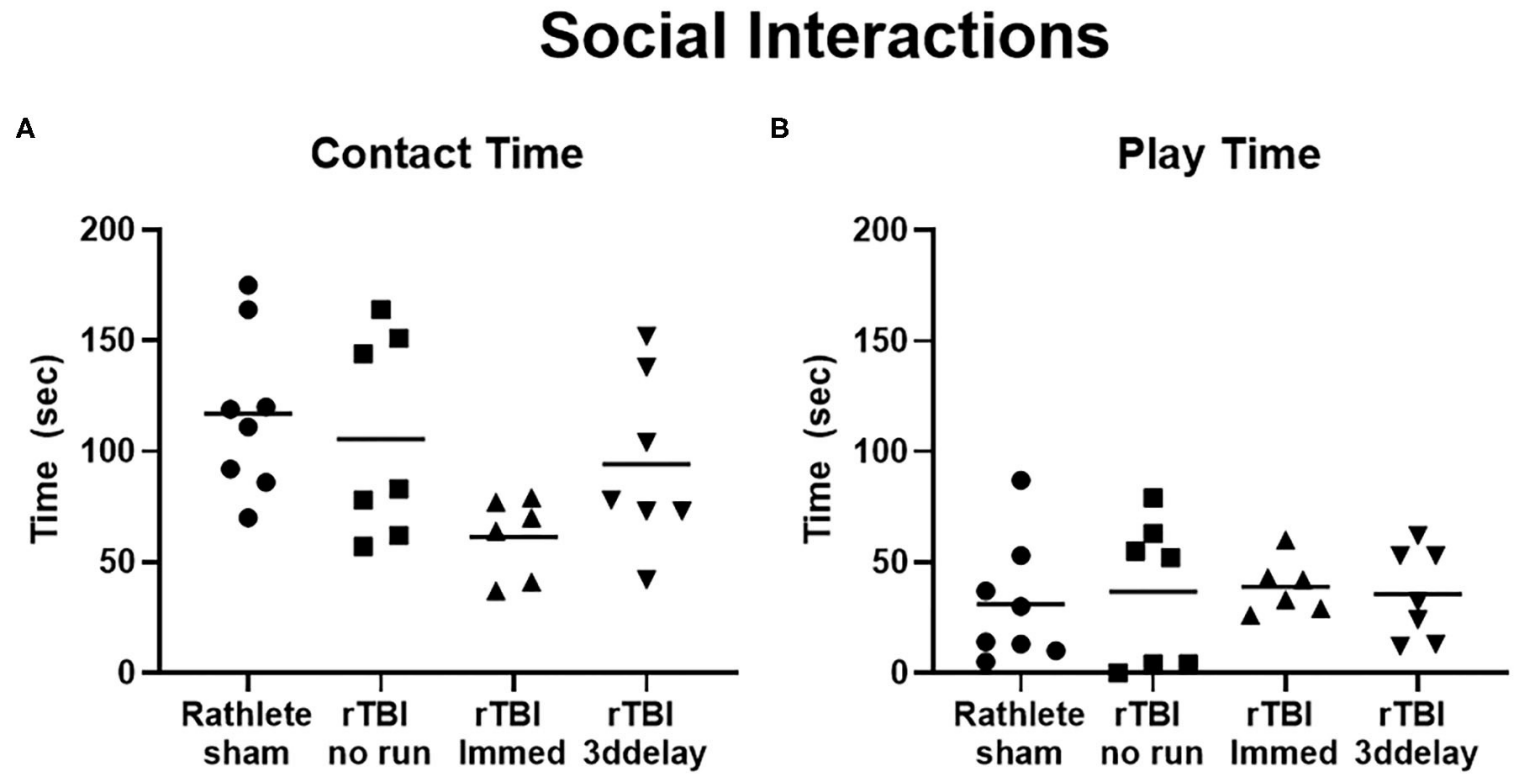
Changes in social interactions. Rathletes were tested for interaction with a novel mate of slightly smaller size and younger age. Rathletes were first placed in 10 gal tank followed by novel mate and interaction was scored for 7.5 min. **(A)** Contacts included grooming, and sniffing. **(B)** Play interactions include chasing and nape attacks.

This test describes the behavior of a rat when interacting with a novel intruder rat and is used to quantify social behaviors. In all rathlete groups, the majority of time spent with an intruder involved contact with the intruder rat (sniffing, grooming); rathlete shams: 117 ± 13 s, rTBI+no run: 106 ± 17 s, rTBI+Immed: 61.3 ± 7 s, rTBI+3ddelay: 94.3 ± 15 s. A one-way ANOVA showed a trend for group differences [*F*_(3, 24)_ = 2.79, *p* = 0.06]. Compared to rathlete shams, rTBI+Immed appeared to engage in contact time to a lesser extent (Figure 9A). Amount of time spent in playful behavior (nape attacks, pinning) were similar and not significant across all groups (rathlete: 31 ± 10 s, rTBI+no run: 37 ± 12, rTBI+Immed: 39 ± 5 s, rTBI+3ddelay: 36 ± 8 s, *F*_(3, 24)_ = 0.12, *p* = 0.95] (Figure 9B). Further, all groups showed significantly more contact time than play time [holm-corrected *t*-tests, *t*_(48)_ = 3.47–5.43 *p*'s < 0.003] except for rTBI+Immed [*t*_(38)_ = 1.23, *p* = 0.22].

#### Memory Impairment

**Figure 10:**
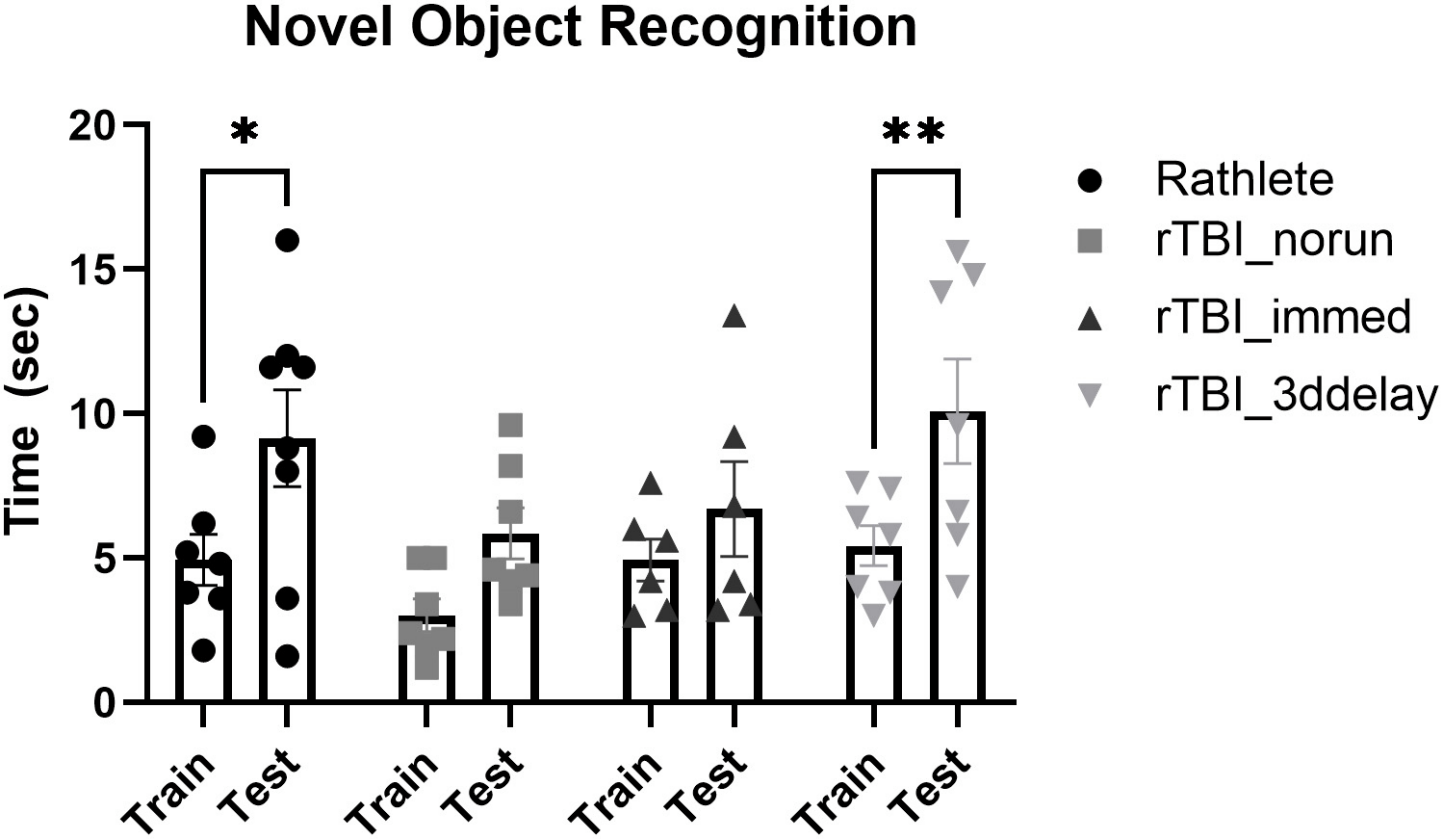
Test of working memory. Novel object recognition task was given to test working memory in rathletes. Rats were habituated to the arena, then given 5 min to interact with 2 identical objects (training day, “train”). Twenty-four hours later, one object was replaced with a novel object (test day, “test”). Time spent interacting with novel object (vs. corresponding object during train trial) was calculated over 5 min interval. Lack of significance suggests memory impairment. *p < 0.05, **p < 0.01.

Working memory was assessed using novel object recognition (Figure 10). All subjects were active in the arena during all days of the task and interacted with the novel object on test day. A two-way repeated measures ANOVA showed no differences between groups [*F*_(3, 24)_ = 2.00, *p* = 0.14] on test day. However, there were differences in ability to recognize novel object (*F*_(3, 24)_ = 26.82, *p* < 0.001). Holm-corrected comparisons found significant increases in time spent with novel object only for rathlete shams (*t*_(23)_ = 3.24, *p* = 0.01) and rTBI+3ddelay (*t*_(23)_ = 3.69, *p* = 0.005), but not for rTBI+norun (*t*_(23)_ = 2.24, *p* = 0.07) nor for rTBI+Immed (*t*_(23)_ = 1.30, *p* = 0.21) indicating cognitive impairment.

During the familiarization trial, the time spent interacting with both objects was recorded. There were differences in object interaction [*F*_(3, 24)_ = 3.64, *p* = 0.03]. rTBI+no run showed significantly less time interacting with both objects (6.01 ± 0.9 s) compared to rathlete shams [11.8 ± 1.3 s*, t*_(24)_ = 3.17, *p* = 0.01]. There was no difference compared to rTBI+Immed [8.9 ± 1.3 s, *t*_(24)_ = 1.33, *p* = 0.35] nor rTBI+3ddelay [10.1 ± 1.2 s, *t*_(24)_ = 0.68, *p* = 0.50].

#### Protein Expression

**Figure 11:**
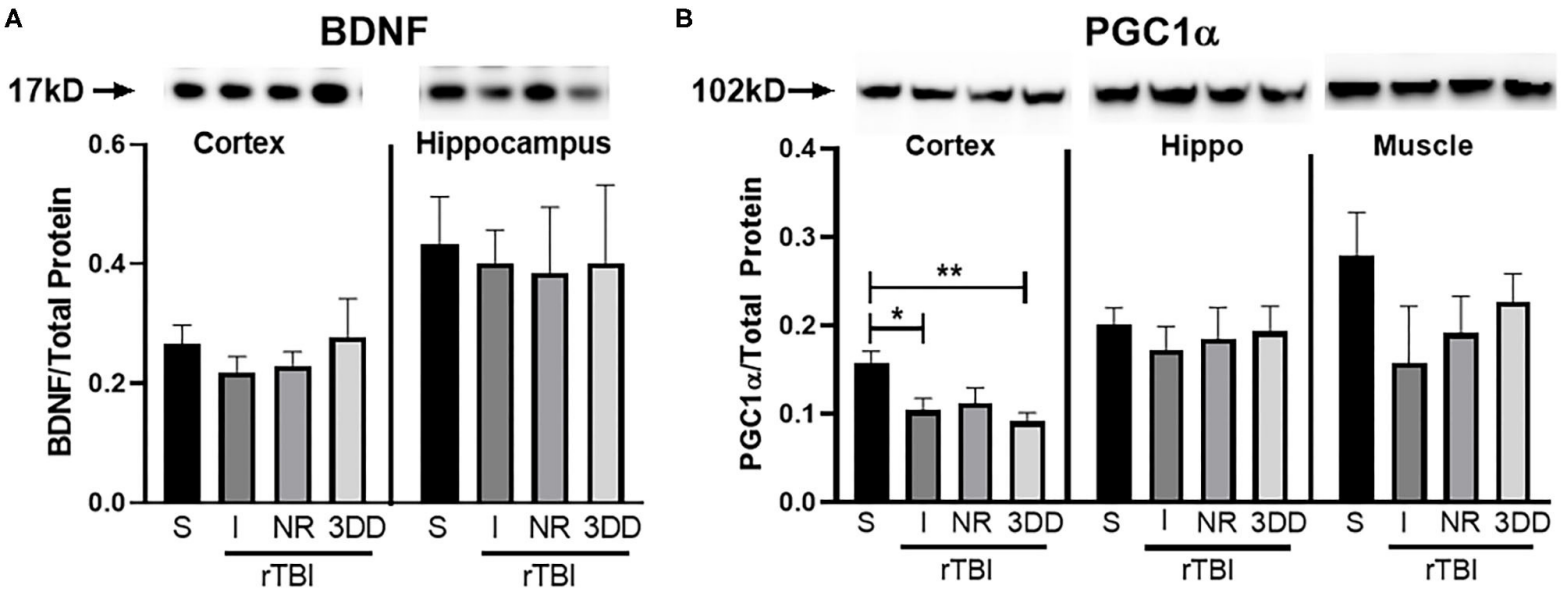
Repeat injury on protein expression in brain and muscle tissue. Parietal lobe (cortex) and hippocampal (hippo) brain tissue, and gastrocnemius (muscle) tissue was isolated from rathlete sham (S) and rTBI rathletes with post-injury groups: no run (NR), run immediately (I), or following a 3 day delay (3DD). Following homogenization, cells were isolated and analyzed using Western blot for **(A)** brain-derived neurotrophic factor (BDNF) and **(B)** peroxisome proliferator-activated gamma coactivator 1-alpha (PGC1α). Density of BDNF and PGC1α protein was normalized to total protein using Ruby and presented as proportion of protein in tissue. *p < 0.05, **p < 0.01.

Changes in BDNF and PGC1α were analyzed (Figure 11). A one-way ANOVA found no significant group differences in BDNF expression for cortex [*F*_(3, 24)_ = 0.49, *p* = 0.69] nor hippocampus [*F*_(3, 24)_ = 0.04, *p* = 0.99] (Figure 11A). There were, however, significant differences in expression of PGC1α in the cortex [*F*_(3, 24)_ = 5.48, *p* = 0.005], but not hippocampus [*F*_(3, 24)_ = 0.19, *p* = 0.90] nor muscle [*F*_(3, 24)_ = 1.23, *p* = 0.32] (Figure 11B). In the cortex, rathlete shams show significantly greater expression of PGC1α compared to rTBI+Immed [*t*_(23)_ = 2.95, *p* = 0.02] and rTBI+3ddelay [*t*_(23)_ = 3.77, *p* = 0.003].

## Discussion

While adolescent athletes are at the highest risk for mTBI and rTBI, the majority of preclinical studies utilize sedentary rats to analyze cognitive recovery. Pre-TBI exercise may change the vulnerability of the adolescent brain to cognitive dysfunction and alter the recovery process. The current study developed a rat model that mirrors adolescent athletes, allowing voluntary running exercise for 20 days. Observations during the last 10 days describe the baseline influence of exercise on neural plasticity and behavior. Results show that exercise alters baseline brain functioning and behavior. Rathlete shams showed increased protein expression of BDNF and PGC1α in the cortex and hippocampus compared to sedentary shams. Similar results were seen with others studying exercise effects on adult mice, showing 4–6 weeks of exercise upregulates neuroprotective and anti-inflammatory markers in the cortex (39, 40).

Aerobic exercise mediates the release of peripheral factors from bone, liver, muscle, and platelets, which cross the blood brain barrier and stimulate various regions of the brain to release BDNF, particularly the hippocampus, promoting enhanced learning and memory (41–43). One rodent study found different patterns of BDNF increase in exercised adults and adolescents compared to their sedentary counterparts, which emphasizes the importance of more pre-clinical studies on the active adolescent (43). Increased BDNF protein expression may provide neuroprotection from brain injury for rathletes through inhibition of neuronal apoptosis and oxidative damage (39, 40, 44). Hippocampal BDNF has also shown to play a role in neuropathic pain. Results from this study support this role as exercised rats showed lower threshold for von Frey filaments, suggesting an increased allodynic response. Rathletes were found to have increased hippocampal BDNF and decreased neurological pain threshold. It may be the peripheral or hippocampal release of BDNF leads to disinhibition of neural firing and downregulation of inward electrochemical gradient of hippocampal neurons that sensitizes individuals to neuropathic pain (45, 46). Coull et al., found that the resulting hyperexcitability of spinal cord neurons lowered the pain threshold in adult rats (45). The increased allodynic response seen in rathletes in the current study may be a result then of the exercise-induced increased BDNF release. The mechanism behind this needs further exploration.

Exercised rathletes spent more time immobile in an open field task. This suggests that adolescent rathletes are more prone to anxiety than sedentary peers. Results shown here may be due to exercise-induced stress (17). In response to stressors, exercised adolescents show enhanced adrenal sensitivity and prolonged release of adrenocorticosterone hormone (ACTH) (47, 48). The adolescent brain undergo changes in response to such stress, particularly in the hippocampus and medial prefrontal cortex. These areas regulate emotionality and may be responsible for the observed increase in anxiety response (17).

Exercised adolescent rats in this study did not show significant improvement in fine motor skills test nor hippocampal-based memory in NOR compared to sedentary peers, a result found by others (43). The NOR task for memory is one that can be cross-generalized across species including human infants and therefore may have clinical relevance to the adolescent and athlete population (49). Previously we have shown that following a short delay, sedentary sham rats perform NOR around 70%; results from this study have replicated those results in rathlete shams. Exercise has no short-term bearing on working memory as sedentary shams and rathlete shams performed at similar levels on recognizing novel object. Looking at the long-term effects of exercise on memory performance may show differences between exercised and sedentary adolescent rats (43). The influence of exercise on the brain following repeat brain injury was addressed in the 2nd experiment in this study.

Prior research has shown that aerobic exercise prior to a moderate-severe TBI protects the adult rodent brain from breakdown of the blood brain barrier, neuroinflammation, motor and memory impairments (40, 50). Whether the same is true for adolescents is still not well-understood. The current study utilized a mild injury model which follows a different recovery timecourse than moderate injuries. Further, this study focused on exercised adolescent rats, corresponding to the population at highest risk for rTBI in humans. The results describe how pre-injury exercise influences recovery. Overall, our results indicate that pre-injury exercise has neuroprotective functions. Rathletes resting post-rTBI (rTBI+norun) did not differ from rathlete shams in motor or cognitive tests for dysfunction. Specifically, behaviors in the open field (anxiety), NOR (memory) and social intruder task were not-significantly different. Previously, we have shown that sedentary adolescent rats showed impairment in the NOR task following rTBI (31). Together, this indicates that pre-injury exercise appears to protect the brain from post-injury impairment.

Assessment of neuropathic pain indicated that all exercise groups show similar sensitivity to von Frey filaments, particularly in the periorbital space contralateral to injury. No significant differences were seen in pain score or threshold to pain, though this may be due to the already high sensitivity (low threshold) in rathletes. Pain responsivity, however was greater in rTBI+3ddelay on PID 7 compared to rathlete shams. Baseline measurements were not performed and thus we were unable to perform pre-injury comparisons. These results are distinct from those reported by others. The von Frey test is most often performed in adult rodents with results showing changes to periorbital threshold following CCI not craniotomy alone (51, 52). The differences observed here may be due to the mild nature of the injury paradigm used in this study. They may also be due to the age of rats tested; adolescent rats are not adults. Adult rat thresholds typically range 8–10 g. In adolescent rathletes tested here, the baseline was much lower, with a maximum of 6 g. By PID7 it seemed that rats anticipated filament and responded quickly. It was harder to approach them unexpectedly and may account for the sensitivity on that day. Limitation should be placed on the results of these data. The allodynia methodology used in this procedure did not include restraint, different from others, and could account for the heightened sensitivity observed. Previous studies have found fluctuations in periorbital thresholds pre-injury and dubbed this “spontaneous trigeminal allodynia” and suggested a genetic component for migraine. In such animals, threshold values ranged from 8 g to <2 g (53, 54). They also showed stable responses of these values over 4 weeks of testing. Rats in that study ranged in weight between 200 and 600 g, though they did not report age or whether weight played role in fluctuating allodynic responses. In the current study, some rats also showed fluctuating thresholds, ranging from 0.4 to 6 g. No additional tests were performed to determine if these subjects also have a genetic component to describe an animal model for migraine.

Chronic inflammatory responses may also relate to the allodynic sensitivity. Oshinsky et al., showed that repeated inflammatory dural stimulation in adult rats decreased von Frey pain threshold that lasted weeks after cessation (55). Exercise has been shown in humans to stimulate release of inflammatory mediators (bradykinin) leading to dural stimulation and is responsible for mechanical pain sensitivity (56–58). Bradykinin has also been linked to secondary brain damage following moderate TBI in mice (59). Levels of inflammatory mediators were not recorded in the current study, but warrant further exploration in regards to rTBI and adolescent rathletes.

The current study also addresses the timing of exercise post-injury on recovery in adolescent athletes, a population that is highly understudied preclinically. Clinical studies on active adolescents and animal studies on sedentary rats indicate that exercise too early or refraining from exercise altogether post-TBI can exacerbate symptoms of concussion (28, 60–62). Delayed exercise, by contrast, has shown to accelerate recovery from concussion in both sedentary rats and exercised adolescents (29, 63). Timing of exercise following rTBI is important in relation to release of lactate from muscles. Lactate can be beneficial, aiding in neurogenesis and angiogenesis (41, 64). Results from the current study focus on adolescent rats active before injury and indicate that no or early (within 3 days) moderate exercise after rTBI had no effect on motor behavior and balance in rathletes. Exercise timing after rTBI did effect cognitive outcomes. Exercise immediately or after a 3 day delay post-rTBI showed cumulative reduction of anxiety compared to rathlete shams and injured rathletes that rested completely. RTBI+Immed rathletes, however, showed impairment in recognition of a novel object, a measure of hippocampal-based memory, and social interactions. Specifically, the rTBI+Immed group showed reductions in social contact time and comparatively more time engaging in play behavior compared to investigative behavior. These results suggest impairment in the proper social interactions of adolescent rats and has been observed by others (65). Social impairment has also been seen in clinical studies analyzing adolescents suffering from TBI (16). We conclude that a brief period of rest prior to returning to exercise is most beneficial in the recovery from rTBI in adolescent rathletes and parallels the graduated clinical return to play protocols already established for adolescent athletes.

Cognitive assessments following concussion play an important role in determining severity of brain injury and treatment. Concussed college athletes have increased metabolic dysfunction as observed by decreased glutamate (Glu) and N-acetyl acetate (NAA), following injury specifically in the dorsolateral prefrontal cortex and premotor cortex (66). These individuals also self-reported migraine-like symptoms (headache, light or noise sensitivity), correlating with decreases in both Glu and NAA in the premotor cortex (66). Importantly, in the absence of physician-scored neurophysiological deficits, it was shown concussed athletes had continued metabolic dysfunction. In the current study, following rTBI injury in rathletes, expression of PGC1α was decreased in the parietal cortex compared to rathlete shams and may represent metabolic dysfunction in this cohort. This decrease was not observed in the hippocampus. Such a region specific difference may be the result of the timing of tissue analysis. Protein analysis was performed 10 days post-injury. It may be that the hippocampus already recovered from injury, while the cortex needs longer to recover. More research is needed on the timeline of mitochondrial and metabolic recovery of adolescent athletes. Current return to play guidelines, allow return to athletic participation following such a “symptom-free” period and clearance from a medical professional. This may not be the best approach as they may still have metabolic depression leaving them vulnerable to a second, cumulative injury. Confirmation of metabolic recovery prior to return to play may be most beneficial to athletes.

### Limitations

There are several limitations of this manuscript. The results of behavioral data shown here with adolescent males were different from studies performed on adult rats, which we believe to be related to age. We believe this to be a strength in the paper as it highlights the uniqueness of the adolescent population, but does provide limitations in interpretations. In the beam walking task we observed a crawling-like behavior of the rats that seem to be absent from adult rats, which made analysis of foot slips difficult. This behavior has not yet been described in other studies, but we believe it to be specific to adolescent rats, particularly males. The battery of behavioral tests was performed over multiple time points, which may affect habituation. In the open field task, we argue that passive behavior is anxiety-like as other studies have shown robust changes in immobility with anxiolytic drugs. Passive behavior in the open field arena is not completely resistant to habituation, however. We analyzed other behaviors (rearing, defecation) that could reflect habituation to the open field arena as well. These need to be taken into context, that behavioral changes in the open field task may not fully represent recovery from rTBI or due to pre-injury exercise. Additionally, we include the von Frey test as a measure of neuropathic pain. It is important to note that this is a mechanosensory test that has been interpreted as neuropathic pain due to behavior of rats, i.e., paw swiping directed at filament. This behavior also has habitual effects; rats learn to anticipate filament presentation and respond more robustly over time. Care was taken to avoid false positives but the potential is present to skew results.

There were some limitations in the groups studied as well. This manuscript did not include a sedentary rTBI group as a control. This was done purposefully. We have previously published results on sedentary rTBI adolescent rats. Our studies and those of others have described post-injury behavioral (28, 31, 65), hormone (67), and metabolic dysfunction (32) in this cohort. As these studies have characterized post-injury recovery profiles in sedentary adolescent rats extensively, we found it unnecessary to replicate this group in the current study. Lastly, this study only included male rats. This was also done purposefully. Clinically, females complain of anxiety and depression to a greater degree than males following TBI. As such, studies including females should include behavioral measures of anxiety and depression. A study by Wright et al., found that only adolescent male rats had short term memory deficits, while only females had depressive-like behavior (68). We are currently performing a follow up study focusing on female adolescent rats including appropriate behavioral measures.

### Summary

This study developed an adolescent rat athlete model to better study post-injury deficits in physically active adolescent rats. As athletes show greatest incidence for mTBI and rTBI, it is imperative that pre-clinical studies include exercise in pre-injury models to best translate results clinically. Results shown here found that male adolescent rats given voluntary aerobic exercise showed differences in cognitive behavior and protein expression of BDNF and PGC1α compared to sedentary male rats. Specifically, these rathletes show heightened anxiety-like behaviors, increased neuropathic pain sensitivity, and greater markers of neuroplasticity and mitochondrial biogenesis. Such differences change the baseline neurobiology. When impacted with rTBI (2 injuries, 24 h apart), those animals given voluntary aerobic exercise training seemed to be protected from some of the cognitive deficits seen in sedentary rats. Specifically, rathletes did not show memory impairment following rTBI, differing from our previous finding of sedentary adolescent male rats (31, 32, 69). This may be due to the pre-injury increase in BDNF protein expression (70, 71). Finally, the current study addressed the effects of early voluntary exercise following rTBI on cognitive and behavioral deficits. Results indicate that complete rest or immediate return-to-activity was detrimental to recovery. These rats show deficits in expression of PC1α indicating potential metabolic disruption 10 days post-injury, and social and memory impairment. Conversely, we show voluntary exercise 3 days after rTBI is beneficial. rTBI+Immed did not show cognitive deficits, though PGC1α depression remained compared to rats that rested 10 days post-injury. These results emphasize the need for immediate concussion evaluation clinically to prevent premature return-to-play and exacerbation of concussion symptoms. Future studies will evaluate similar effects on females and changes in biochemistry of adolescent rathletes.

## Data Availability Statement

The original contributions presented in the study are included in the article/supplementary materials, further inquiries can be directed to the corresponding author/s.

## Ethics Statement

The animal study was reviewed and approved by The UCLA Chancellor's Animal Research Committee and National Institute of Health Guide for the Care and Use of Laboratory Animals.

## Author Contributions

CG, TG, MF, and MP conceived the ideas presented and developed the behavioral paradigms used in this study. LF and RS performed the experiments and analyses. LF wrote the initial draft of the manuscript. All authors discussed the results and contributed to the final manuscript.

## Conflict of Interest

The authors declare that the research was conducted in the absence of any commercial or financial relationships that could be construed as a potential conflict of interest.
